# Cushing's syndrome: Consequences of late diagnosis after bariatric surgery

**DOI:** 10.1002/ccr3.2694

**Published:** 2020-02-05

**Authors:** Jorge Pedro, Sandra Belo, Vanessa Guerreiro, Maria João Ferreira, Daniela Salazar, Cláudia Costa, César Esteves, Josué Pereira, Paula Freitas, Davide Carvalho

**Affiliations:** ^1^ Department of Endocrinology, Diabetes and Metabolism Centro Hospitalar Universitário de São João Porto Portugal; ^2^ Faculty of Medicine Universidade do Porto Porto Portugal; ^3^ Instituto de Investigação e Inovação em Saúde (i3S) Universidade do Porto Porto Portugal; ^4^ Department of Endocrinology Instituto Português de Oncologia do Porto Porto Portugal; ^5^ Department of Neurosurgery Centro Hospitalar Universitário de São João Porto Portugal

**Keywords:** bariatric surgery, Cushing's syndrome, osteoporosis

## Abstract

Prior to bariatric surgery, endocrine causes of obesity must be excluded. The diagnosis of osteoporosis in a male requires the study of secondary causes of this condition. The diagnostic delay of Cushing's syndrome may have irreversible consequences.

## Graphical abstract

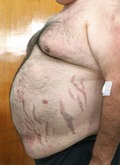

## 1 INTRODUCTION

A case of a 53‐year‐old male submitted to two different bariatric surgeries without having any preoperative endocrine study. Afterward, the patient was diagnosed with Cushing's syndrome and severe osteoporosis. Timely diagnosis and treatment of both conditions could have prevented the surgical procedures and resulting comorbidities.

Bariatric surgery (BS) is indicated for patients with severe obesity,^1^ and this condition is a very common clinical manifestation of Cushing's syndrome (CS).^2^ Prior to the procedure, all patients should undergo preoperative evaluation for obesity‐related comorbidities and causes of obesity. If suspected clinically, screening for CS is recommended with 1 mg overnight dexamethasone test, 24‐hour urinary free cortisol, or midnight salivary cortisol.^1^

Cushing's syndrome comprises a large group of signs and symptoms that reflect prolonged and inappropriately high exposure of tissue to glucocorticoids. Although the most common cause of CS is iatrogenic, derived from prescription corticosteroids, endogenous CS could also account for a proportion of cases, being deemed as an uncommon disorder.^2^ If the cause is endogenous, Cushing's disease accounts for most of the cases.^3^ Osteoporosis affects approximately 20%‐40% of patients with CS, which is manifestly less prevalent than other comorbidities. This may result in diagnostic and treatment delay of this condition.^4^ It is also important to bear in mind that all men diagnosed with osteoporosis should be evaluated for secondary causes of bone loss.^5^

The aim of this case report is to emphasize that it is essential, prior to the surgical treatment of obesity, that the patient is evaluated for secondary causes of obesity. We also intend to highlight osteoporosis as a serious complication of CS and the need to screen for the condition of all patients with hypercortisolism.

## 2 CASE HISTORY

**Figure 1:**
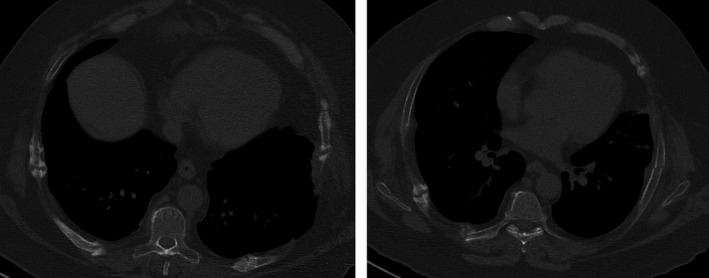
High‐resolution chest CT with evident fractures of costal arches

A 53‐year‐old male referred to the Endocrinology outpatient department after undergoing BS. He was professionally active as construction worker and his medical history was relevant to obesity (BMI 45.7 kg/m^2^), hypertension, chronic pain, a 70‐pack‐year smoking history and two BS, adjustable gastric banding (12 years prior), and Roux‐en‐Y gastric bypass (1 year earlier). His current medication included lisinopril, acemetacin, tapentadol, and duloxetine. One month earlier, the patient was evaluated in a pain management clinic. He had complaints of chest pain that intensified with inspiration, moving, or coughing. He denied any history of previous thoracic trauma. A high‐resolution chest CT scan was requested. The image revealed bilateral fractures of almost all costal arches (Figure 1).

**Figure 2:**
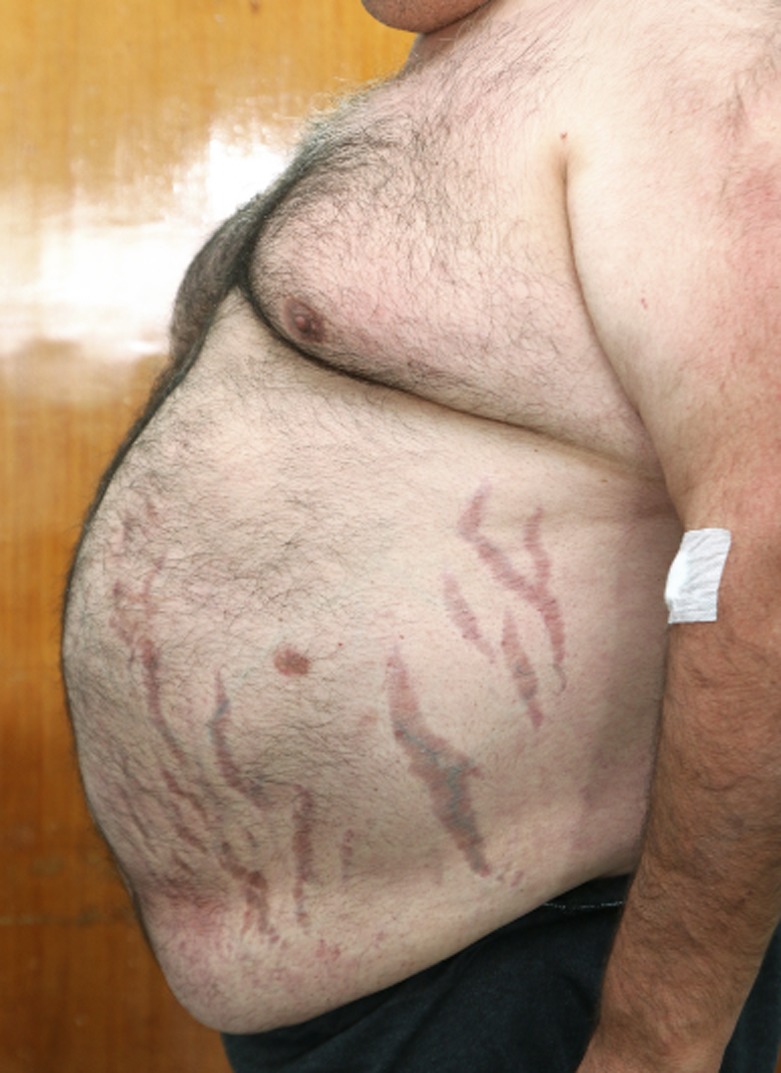
Centripetal obesity with vinous striae

**Figure 3:**
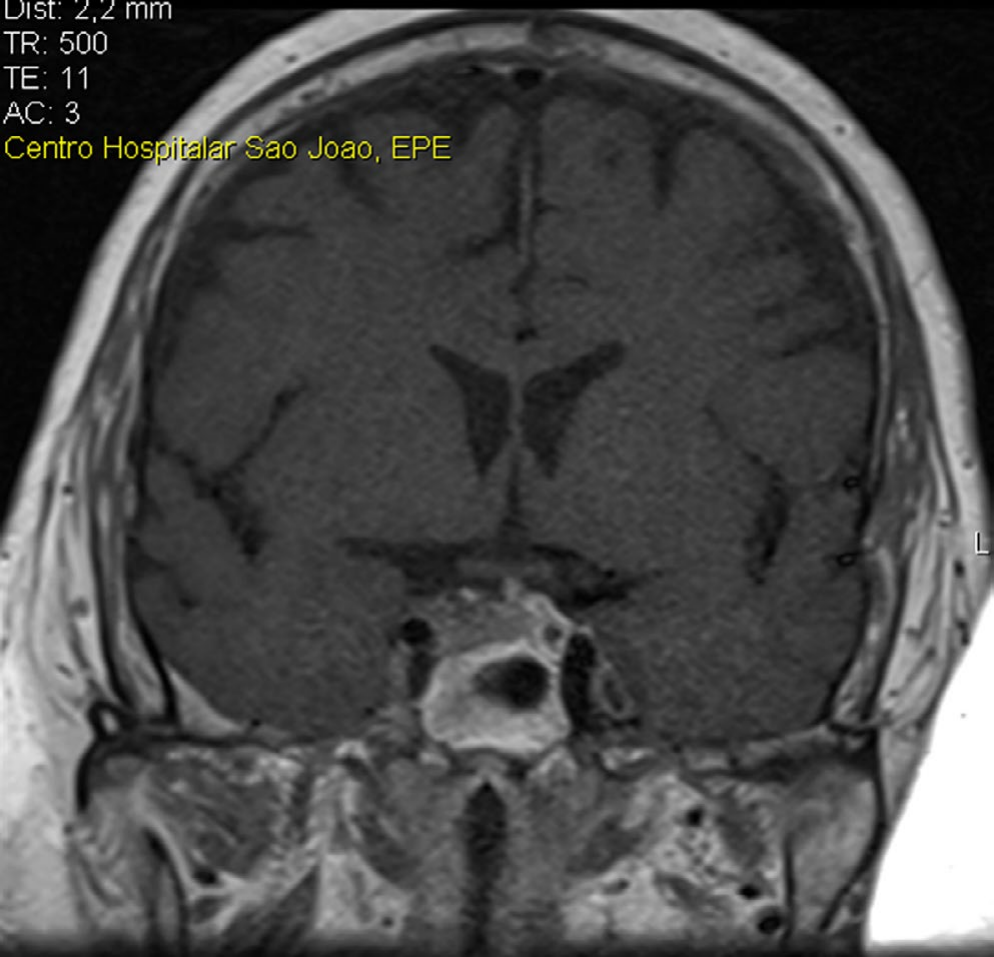
Pituitary Magnetic Resonance showing a macroadenoma with 9.6 × 22.3 mm

**Figure 4:**
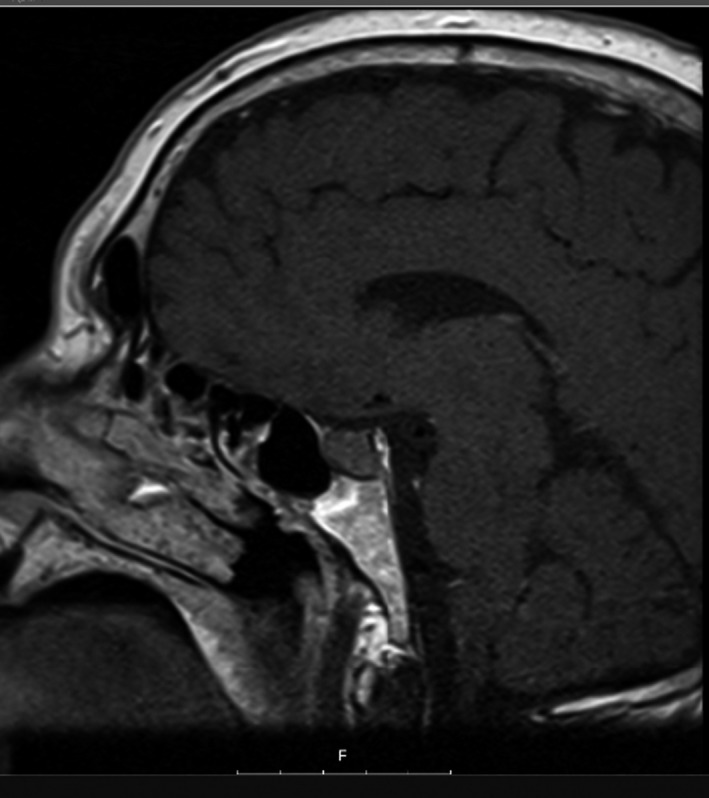
Pituitary Magnetic Resonance showing a macroadenoma with 9.6 × 22.3 mm

During the outpatient consultation, our observation revealed he was submitted to an adjustable gastric banding 11 years ago with a significant weight loss but with weight regain. Consequently, he was then submitted to Roux‐en‐Y gastric bypass. In the postoperative period of this surgery, the use of antibiotics was necessary to heal an infection of the surgical wound. No other postoperative complications were described. The patient also stated that, at the time of the second surgery, in addition to central obesity, he already had rib cage pain, facial plethora, and easy bruising. No endocrine cause for obesity was studied prior to both surgical procedures. The patient complained of bone pain and significant limitation in gait and daily living activities for several years. Physical examination revealed several signs suggestive of CS: facial plethora, centripetal obesity (Figure 2), dorsocervical and supraclavicular fat pads, vinous striae (Figure 2), easy bruising, acanthosis nigricans, and proximal myopathy. His endocrine evaluation was consistent with the diagnosis of Cushing's disease and the Pituitary Magnetic Resonance demonstrated a macroadenoma with 9.6 × 22.3 mm (Figures 3 and 4) with cavernous sinus invasion. The patient had no complications due to a large compressing macroadenoma. The complementary study also revealed osteoporosis.

## 3 INVESTIGATION

Chest CT scan revealed bilateral fractures of almost all costal arches (Figure 1).

Bone densitometry of the lumbar spine and femur showed a T‐score of −3.4, compatible with osteoporosis.

Laboratory results were consistent with CS and dyslipidemia (Table 1). Other laboratory findings were unremarkable, including other hormones produced by the pituitary gland.

**Table 1:** Biochemical results table

Parameter	Value
Total cholesterol	224 mg/dL (<200)
HDL cholesterol	43 mg/dL (>60)
Triglycerides	159 mg/dL (<150)
LDL cholesterol	149 mg/dL (<130)
24 h urinary free cortisol	686.6 µg/day (36‐137)
Plasma cortisol after 1‐mg overnight dexamethasone suppression test	20 µg/dL (<1.8)
ACTH	46.3 ρg/mL (10‐60)
Plasma cortisol after high‐dose dexamethasone suppression test (Liddle test)	
Baseline	20 µg/dL
48 h	2.4 µg/dL
Prolactin	5.3 ng/mL (4‐15.2)

Pituitary Magnetic Resonance demonstrated a macroadenoma with 9.6 × 22.3 mm (Figures 3 and 4) with cavernous sinus invasion.

## 4 TREATMENT

Initially, pharmacological treatment for osteoporosis was initiated with calcium carbonate 500 mg bid, cholecalciferol 400 UI bid, and ibandronic acid 150 mg monthly. Atorvastatin was also prescribed to treat dyslipidemia. The patient underwent transsphenoidal surgery with subtotal removal of the lesion.

Recently, teriparatide 20 mcg id was initiated and ibandronic acid was suspended.

## 5 OUTCOME AND FOLLOW‐UP

Currently, 1 year after surgery, the patient maintains regular follow‐up at the endocrinology clinic and he is analytically free of hypercortisolism, clinically shows a slight improvement in gait and has already lost about 10 kg.

## 6 DISCUSSION

The contribution of bariatric surgery to the treatment of obesity is undeniable.^6^ However, according to most current guidelines, it is essential to make a preoperative evaluation in order to exclude secondary causes of this pathology.^1^ We often come across patients who are evaluated postoperatively without ever having been preoperatively. This case exemplifies that. This patient had several stigmas of the disease that were not recognized until the patient was evaluated by Endocrinology. This highlights the extreme importance of physical examination. Several signs of CS may be present: rounding of the face (moon face), a pad of fatty tissue between the shoulders and neck (buffalo hump), thin skin with bruises and stretch marks, acne, or muscle weakness. This delay in the diagnosis of the disease led to the development of some consequences of CS that could be potentially avoidable, many of which irreversible and with high impact in the daily living activities of the patient.^7^

Osteoporosis is often associated with CS and, as stipulated in several recommendations, it is indicated to screen for the condition in these patients.^8^ However, it is overlooked at the expense of other comorbidities. In this context, osteoporosis occurs through different mechanisms: decreased intestinal calcium absorption, bone formation and renal calcium reabsorption, and increased bone resorption.^9^ On the contrary, all men diagnosed with osteoporosis should be evaluated for secondary causes of bone loss,^5^ such us CS. Therefore, it is essential that clinicians be aware of the coexistence of both entities and their relationship in order to improve the outcome of the patients.

## CONFLICT OF INTEREST

The authors declare that there is no conflict of interest that could be perceived as prejudicing the impartiality of this case report.

## AUTHOR CONTRIBUTION

JP: wrote this case report and was involved in the patient care. SB, VG, CE, JP, and PF: involved in the patient care and revised the draft. MJF, DS, and CC: helped in the writing of this case report. DC: revised the draft.

## PATIENT CONSENT

Written informed consent has been obtained from the patient.

## References

[1] Mechanick JI , Youdim A , Jones DB , et al. Clinical practice guidelines for the perioperative nutritional, metabolic, and nonsurgical support of the bariatric surgery patient‐2013 update: Cosponsored by American association of clinical endocrinologists, the obesity society, and American society. Obesity. 2013;21(SUPPL. 1):159‐191. 23537696 10.1016/j.soard.2012.12.010

[2] Nieman LK , Biller BMK , Findling JW , et al. The diagnosis of Cushing's syndrome: an endocrine society clinical practice guideline. J Clin Endocrinol Metabol. 2008;93(May):1526‐1540. 10.1210/jc.2008-0125 PMC2386281 18334580

[3] Sharma ST , Nieman LK , Feelders RA . Cushing's syndrome: epidemiology and developments in disease management. Clin Epidemiol. 2015;7:281‐293. 25945066 10.2147/CLEP.S44336 PMC4407747

[4] Valassi E , Santos A , Yaneva M , et al. The European Registry on Cushing's syndrome: 2‐year experience. Baseline demographic and clinical characteristics. Eur J Endocrinol. 2011;165(3):383‐392. 21715416 10.1530/EJE-11-0272

[5] Watts NB , Adler RA , Bilezikian JP , et al. Osteoporosis in men: an endocrine society clinical practice guideline. J Clin Endocrinol Metab. 2012;25(June):1802‐1822. 10.1210/jc.2011-3045 22675062

[6] Kissler HJ , Settmacher U . Bariatric surgery to treat obesity. Semin Nephrol. 2013;33(1):75‐89. 23374896 10.1016/j.semnephrol.2012.12.004

[7] Lamos EM , Munir KM . Cushing disease: highlighting the importance of early diagnosis for both de novo and recurrent disease in light of evolving treatment patterns. Endocr Pract. 2014;20(9):945‐955. 25100372 10.4158/EP14068.RA

[8] Nieman LK , Biller BMK , Findling JW , et al. Treatment of Cushing's syndrome: an endocrine society clinical practice guideline. J Clin Endocrinol Metabol. 2015;100(August):2807‐2831. 10.1210/jc.2015-1818 PMC4525003 26222757

[9] Kaltsas G , Makras P . Skeletal diseases in Cushing's syndrome: osteoporosis versus arthropathy. Neuroendocrinology. 2010;92(suppl 1):60‐64. 20829620 10.1159/000314298

